# Thrombocytopenia After Aortic Valve Replacement: Comparison Between Sutureless Perceval S Valve and Perimount Magna Ease Bioprosthesis

**DOI:** 10.21470/1678-9741-2017-0157

**Published:** 2018

**Authors:** Syed Saleem Mujtaba, Simon Ledingham, Asif Raza Shah, Stephan Schueler, Stephen Clark, Thasee Pillay

**Affiliations:** 1 Department of Cardiothoracic Surgery, Freeman Hospital, Freeman Road, United Kingdom of Great Britain and Northern Ireland.

**Keywords:** Aortic Valve/Surgery, Thrombocytopenia, Bioprosthesis/ Adverse Effects, Heart Valve Prosthesis Implantation/Adverse Effects

## Abstract

**Introduction:**

The incidence of postoperative thrombocytopenia after aortic valve replacement (AVR) with the Perceval S Sutureless bioprosthesis remains unclear. The aim of this study was to report thrombocytopenia associated with the use of sutureless AVR.

**Methods:**

The data was collected retrospectively for patients who had isolated AVR with sutureless Perceval S valve (Group A: 72 patients) and was compared with patients who underwent isolated sutured AVR with Perimount Magna Ease Bioprosthesis (Group B: 101 patients) in our institution between June 2014 and January 2017.

**Results:**

Cardiopulmonary bypass and cross-clamp time were significantly shorter in group A. Maximum drop in platelet count was 58% mean (day 2.3) in group A *versus* 44% mean (day 1.7) in group B (*P*=0.0001). Absolute platelet count on postoperative day 1-6 in group A was significantly less than in group B (*P*≤0.05). Platelet count recovered to preoperative value in 44% patients in group B *versus* only in 26% patients in group A at discharge (*P*=0.018). Moderate thrombocytopenia occurs more often in group A (41% *vs.* 26%) (*P*=0.008) while severe thrombocytopenia (<50 x 10^9^) was observed in 6% in group A but never in group B. Platelets (*P*=0.007) and packed red blood cells (*P*=0.009) transfusion was significantly higher in the group A.

**Conclusion:**

The implantation of sutureless Perceval aortic valves was associated with a significant drop in platelet count postoperatively with slow recovery and higher platelets and packed red blood cells transfusion requirements. A prospective randomised trial is needed to confirm our findings.

**Table:** 

Abbreviations, acronyms & symbols	
AVR	= Aortic valve replacement
CPB	= Cardiopulmonary bypass
ECC	= Extracorporeal circulation
POD	= Postoperative day
TAVI	= Transcatheter aortic valve implantation

## INTRODUCTION

Surgical aortic valve replacement (AVR) still represents the gold standard in patients with severe aortic valve stenosis^[1]^. Owing to increasing age of the patient population in the Western world, there has been an increase in the prevalence of patients with valvular heart disease eligible for AVR^[2]^. Given the increasing number of co-morbidities and the increasing age of patients, a tendency has emerged to use biological valve implants thus avoiding the need for long-term anticoagulation therapy. Although the concept of transcatheter aortic valve implantation (TAVI) appears attractive, the calcified aortic valve is not removed during this procedure. Therefore, paravalvular leakage remains an important issue with this technique^[3]^. Other important concerns are access site-related problems and device malpositioning.

The recent introduction of sutureless bioprostheses may offer an additional tool in the therapeutic armamentarium as these valves do not need to be sutured into place resulting in shorter cross-clamp and cardiopulmonary bypass (CPB) durations which may be beneficial in older patients with co-morbid conditions. Moreover, due to the absence of a sewing ring, these valves exhibit favourable hemodynamic properties. Excellent outcomes have been demonstrated with sutureless AVR in minimally invasive surgical setting^[4]^.

We noticed that sutureless valves are associated with greater drops in postoperative platelet counts compared to sutured valves. Albacker^[5]^ reported this phenomenon with sutureless AVR and the aim of this study was to investigate this observation and try to find an explanation for its occurrence.

## METHODS

### Patients

It is a retrospective, observational study. All the patients who underwent isolated sutureless Perceval AVR between June 2014 and November 2016 were compared with patients who had isolated AVR with Carpentier-Edwards Perimount Magna Ease valve between June 2015 and January 2017. Any non-isolated AVR cases *i.e*.: coronary artery bypass grafting, mitral and tricuspid valve surgery, myomectomy, ascending aorta surgery were excluded. Moreover, all the redo, emergency and infective endocarditis patients were excluded.

All the sutureless AVRs were performed by the six different surgeons in our institution during that period. Data was collected on all the patients who underwent sutured AVR by the same surgeons during the same time period (101 patients) as a control group.

A total of 72 patients received isolated Perceval bioprosthesis and 101 patients had Perimount Magna Ease valve. The Percevel S valve sizes were small, medium, large and extra-large. For statistical purposes they were taken as equivalent to 19-21, 23, 25 and 27 mm Perimount Magna Ease sizes, respectively.

Severe thrombocytopenia was defined as a platelet count <50x10^9^/Lit during the postoperative period. The platelet count was determined preoperatively, day of surgery and every day until day 5 in the postoperative period. In-hospital mortality was defined as any death occurring within first 30 days after surgery. Aspirin and clopidogrel was discontinued 7 days before surgery and warfarin was stopped 5 days before operation.

### Surgical Approach

A complete median sternotomy was performed and extracorporeal circulation (ECC) with moderate hypothermia (34ºC) was employed in every patient. Antegrade cold blood cardioplegia was administered after application of cross clamp. The choice of prosthesis implanted was made by the surgeon. Perioperative transesophageal echocardiogram was performed routinely to confirm correct position of the valve, exclude any paravalvular leak and evaluate valve hemodynamics.

The Perimount Magna Ease valve was rinsed with normal saline for 3 minutes before implantation while Perceval valve does not require any rinsing before implantation. Perimount Magna Ease valves were intra implanted with interrupted or semi continuous 2/0 prolene sutures.

The Perceval valve is a surgical bioprosthetic heart valve consists of glutaraldehyde-fixed bovine pericardium treated with homocysteic acid in order to remove the free aldehyde residues and prevent the calcification process. It is fixed in a metal cage made up of an alloy of nickel and titanium, known as nitinol.

Three 4/0 polypropylene guiding sutures were passed at the nadir of the aortic annulus. An appropriately sized prosthesis was collapsed in a side table and placed into the manufacturer's holder. The three guiding sutures were passed through the three green holes arising from the annular ring of the prosthesis, which was consequently seated on the debrided annulus. Once the delivery system is in position, the stent is deployed by turning the release screw and leaving the valve in place. The delivery system and the guiding sutures are removed. The field was rinsed with warm saline, and the prosthesis was dilated at four atmospheres for 30 seconds.

On first postoperative day, patients were started on aspirin 150 mg orally and low molecular weight heparin subcutaneously for deep vein thrombosis prophylaxis.

### Statistical Analysis

All data has been extracted from the Dendrite PATS CS2010F SCTS v4.1.2 database. Date of data extract was 25.07.17 to cover the period from 01.06.2014 to 31.01.17. Analysis was performed using MS Excel. Numerical values were compared using an independent t-Test, with a two-tailed distribution assuming unequal variances. Categorical variables were compared using Chi Square χ2 analysis.

## RESULTS

The preoperative characteristics of patients are depicted in Tables 1 and 2. There was no difference between Group A and B regarding history of preoperative renal impairment or pulmonary disease. Group A has more female patients (A=52% *vs.* B=31%; *P*≤0.004), patients with hypertension (79% *vs.* 60%; *P*=0.01), neurological dysfunction (17% *vs*. 7%; *P*=0.05) and angina (54% vs. 37%; *P*=0.03). In group A, patients were older (range: 54-84, mean 74 *vs*. 47-86, mean 70 *vs*. range 34-91, mean 71; *P*=0.001) and with higher logistic EuroSCORE (3.07 *vs*. 1.87; *P*=0.001).

**Table 1:** Preoperative summary.

	Perceval		Perimount		Chi-squared Test
Total	n=72	%	n=101	%	*P*
Gender (male/female)	34/38	47/52%	69/31	69/31%	0.004
Cigarette smoking history	3	4%	11	11%	0.106
History of hypertension	57	79%	60	60%	0.01
Renal disease at time of surgery	__	__	1	1%	0.40
History of pulmonary disease (i.e: COPD, asthma)	16	22%	19	19%	0.61
History of neurological disease (*i.e*.: TIA, CVA)	12	17%	7	7%	0.05
Angina Status pre-surgery	39	54%	37	37%	0.03
0. No angina	33	46%	63	88%	
1. No limitation of physical activity	16	22%	18	25%	
2. Slight limitation of ordinary activity	11	15%	11	15%	
3. Marked limitation of ordinary physical activity	10	14%	6	8%	
4. Symptoms at rest or minimal activity	2	3%	2	3%	
Dyspnoea status pre-surgery	65	90%	82	82%	0.13
1. No limitation of physical activity	7	10%	18	18%	
2. Slight limitation of ordinary physical activity	23	32%	39	39%	
3. Marked limitation of ordinary physical activity	41	57%	40	40%	
4. Symptoms at rest or minimal activity	1	1%	3	3%	
History of diabetes mellitus	13	18%	20	20%	0.75
Preoperative heart rhythm					
0. Sinus rhythm	55	76%	84	87%	
1. Atrial fibrillation/flutter	16	22%	12	12%	0.07
2. Complete heart block/pacing	1	1%	__	__	0.24
3. Other abnormal rhythm	__	__	1	1.0%	
Ejection fraction category					
1. Good (LVEF > 50%)	58	81%	84	84%	
2. Fair (LVEF 30-50%)	12	17%	15	15%	0.77
3. Poor (LVEF < 30%)	2	3%	1	1%	0.38

COPD=chronic obstructive pulmonary disease; CVA=cerebrovascular accident; LVEF=left ventricular ejection fraction; TIA=transient ischemic attack

**Table 2:** Preoperative summary (continued).

	Perceval			Perimount			t-Test
	Range	Mean	Median	Range	Mean	Median	Two-Sample Assuming Unequal Variances
Age of patients at time of procedure	54-84	74	75	47-86	70	70.5	<0.001
Logistic EuroSCORE comparison	0.53-18.89	3.07	2.41	0-7.57	1.87	1.44	0.001
Height (cm)	140-185	164.1	163	138-185	167.3	169	0.029
Weight (kg)	40.3-158	82.2	80.1	50-181.6	84.90	81.1	0.387

Greater number of L and XL size valves was implanted in Perceval valve group, and of 23 and 25 mm prosthesis in Perimount Magna Ease group (Table 3).

**Table 3:** Proportion of different valve sizes.

Perceval valve sizes	N	%	Perimount Magna valve sizes	N	%
			19	1	1
Small	8	11	21	20	11
Medium	20	28	23	42	42
Large	22	31	25	29	29
X large	22	31	27	10	18
			29	__	__
Total	72			101	

When Perceval valve sizes S, M, L and XL were compared with their counterpart Perimount Magna Ease valve sizes, there was more significant thrombocytopenia in Perceval group (Table 4). Haematology data is depicted in Tables 5, 6 and 7. Among the Perceval valve group, 5 (6%) patients had severe thrombocytopenia. Their peak reduction was on day 3 with 58% of their preoperative platelet value. While in Perimount Magna group none of them had severe thrombocytopenia. Their peak reduction was on day 2 with 44% of preoperative value which is significantly less then Perceval valve group. Perceval valve group required more blood (1.5 *vs*. 0.7 units) (*P*=0.009) and platelet (0.3 *vs*. 0.1 platelets pools; *P*=0.007) transfusion. No significant difference in absolute platelet count between two groups on preoperative and operation days was observed. Platelet count was significantly reduced in Perceval group from day 1-6 and 20% patients came back to preoperative level at discharge. Forty-four percent of Perimount patients came back to preoperative value at the time of discharge. Forty-one percent of Perimount group had moderate thrombocytopenia while this value was 26% in Perceval valve group.

**Table 4:** Comparison of different sizes of valves in respect of drop in platelet count %.

Perceval valve Size %	Perimount Magna valve Size %	*P* value
Small 66.11%	19-21 mm 48.36%	0.0001
Medium 56.28%	23 mm 43.25%	0.0003
Large 59.41%	25 mm 44.85%	0.0003
X large 56.89%	27-29 mm 39.3%	0.0005

**Table 5:** Haematology data.

	Perceval valve N=72	Perimount Magna N=101	*P*
Maximum drop in platelet count: "which day"	Mean 2.3 Median 2 Range 0-5	Mean 1.7 Median 2 Range 0-6	0.0005
Maximum drop in platelet count: "what %"	Mean 58.0% Median 58.3% Range 35-84%	Mean 44.3% Median 45.0% Range 14-64%	<0.0001
Blood transfusion: units	Mean 1.5 Median 0.5 Range 0-10	Mean 0.7 Median 0 Range 0-8	0.0091
Platelet transfusion: pool of platelets	Mean 0.3 Median 0 Range 0-4	Mean 0.1 Median 0 Range 0-2	0.0075
FFP Transfusion: units	Mean 0.2 Median 0 Range 0-4	Mean 0.1 Median 0 Range 0-3	0.21

**Table 6:** Haematology data (continued).

Patients having postoperative thrombocytopenia	Perimount Magna valve	Perceval valve	*P* value
Moderate thrombocytopenia: <100 x 109	N=26 (26%)	N=34 (41%)	0.008
Severe thrombocytopenia: <50 x 109	__	N=5 (6%)	
Patients whose platelet level returned to preoperative level following surgery/did not return to preoperative	Perimount Magna valve N=44/57 (44/56%)	Perceval valve N=20/56 (26/74%)	0.018

**Table 7:** Absolute platelet count on preoperative, operative and postoperative days.

Platelet count on days	Perimount Magna valve	Perceval valve	*P* value
Preoperative platelet count	244	239.8	=0.73
Operation day platelet count	158.6	144.6	=0.07
Postoperative day 1 platelet count	158.7	132.8	=0.0007
Postoperative day 2 platelet count	145.9	112.8	<0.0001
Postoperative day 3 platelet count	158.4	111.4	<0.0001
Postoperative day 4 platelet count	183.5	130.0	<0.0001
Postoperative day 5 platelet count	216.4	157.5	<0.0001
Postoperative day 6 platelet count	260.3	174.4	<0.0001
Postoperative day 7 platelet count	254.6	205.7	=0.23

Intraoperative and early postoperative variables are summarized in Tables 8 and 9. Cross clamp (range 23-76 min, mean 39 min *vs*. 24-133 min, mean 54 min; *P*=0.001)) and bypass time (range 25-119 min, mean 59 min *vs*. range 19-175 min, mean 71 min; *P*=0.001) were shorter in isolated Perceval valve group (Group A1) compared to isolated conventional valve group (Group B1).

**Table 8:** Intraoperative and early postoperative data.

	Perceval			Perimount			t-Test: Two-Sample Assuming Unequal Variances
	Range	Mean	Median	Range	Mean	Median	*P*
Cumulative cross-clamp time (min)	23-76	39.2	38	24-133	54.1	48.5	<0.001
Cumulative bypass time	25-119	59.3	56.5	19-175	71.6	63.5	<0.001
Post operative blood loss @ 12 hours	15-2000	304	212.5	100-2000	359.7	300	0.197
ITU stay in days	1-13	3.2	2	1-17	1.9	1	0.005

ITU=intensive care unit

**Table 9:** Intraoperative and early postoperative data (continued).

	Perceval		Perimount		Chi-squared Test
Total	n=72	%	n=100	%	*P*
Reoperation for bleeding, tamponade	2	2.78%	2	2.00%	0.738
Patient status at discharge (mortality)	1	1.39%	__	0.00%	0.237

Perceval valve patients spent more time in ICU compared to Perimount Magna patients (3.2 days *vs*. 1.9 days; *P*=0.005). No significant difference in postoperative neurological dysfunction, renal impairment, atrial fibrillation, permanent pacemaker requirement or mortality could be observed.

## DISCUSSION

Our retrospective study showed that there is a relationship between Perceval S valve implantation and severe thrombocytopenia. Severe thrombocytopenia in our study was not associated with higher mortality and morbidity, and none of these deaths was related to severe thrombocytopenia.

Sánchez et al.^[6]^ compared the incidence of thrombocytopenia after AVR with Perceval S Sutureless Bioprosthesis (n=27) and Mitroflow prostheses (n=50). The incidence of severe thrombocytopenia was significantly higher (*P*=0.046) in Perceval S patients than in Mitroflow patients. The platelet count recovered in all patients with severe thrombocytopenia.

The biological structure of the Perceval S valve and Freedom Solo stentless bioprosthesis are very similar^[6]^.

Hilker et al.^[7]^ compared the postoperative courses of platelet counts in patients having had AVR with stentless prostheses (Sorin Biomedica Freedom Solo [SOLO]) or stented prostheses (Carpentier Edwards Perimount [PM]). A higher occurrence of platelet levels below 100 Gpt/l between the second and the fifth postoperative day (POD) was found in the SOLO-group (71.9%) compared with the other biological substitute PM (36.6%).

Miceli et al.^[8]^ evaluated the postoperative evolution of platelet count and function after AVR in 116 patients undergoing isolated stentless biological AVR with Freedom Solo and compared with 206 patients who received stented biological valves. Freedom Solo implantation was associated with a higher incidence of thrombocytopenia compared with the control group (24.1% *vs*. 4.4%, *P*<0.0001).

Piccardo et al.^[9]^ also studied the incidence and clinical impact of thrombocytopenia in patients receiving AVR with Freedom Solo bioprosthesis and Carpentier-Edwards Perimount pericardial prosthesis. They reported that severe thrombocytopenia occurred in 25% and 3% of patients with Freedom Solo and Perimount bioprostheses, respectively (*P*<0.0001).

Yerebakan et al.^[10]^ compared platelet counts within 2 weeks after implantation of either a stentless (Sorin Freedom Solo) or a stented (Sorin Mitroflow) bovine pericardial bioprosthesis. In the Mitroflow group, the mean platelet count moderately dropped to a minimum of 60% of the initial value on 3^rd^ POD and fully recovered on 8^th^ POD. In the Freedom Solo group, platelet loss was significantly more severe (minimum relative value 25% on 4^th^ POD) with no recovery during follow-up. However, there were no other complications reported in association with thrombocytopenia related to the use of these Freedom Solo valves and the phenomenon was transient and resolved without clinical consequences or hemodynamic dysfunction^[9,10]^.

Platelet count decrease has also been reported after percutaneous coronary intervention^[11]^. Gallet et al.^[12]^ studied the effect of transcatheter (via femoral artery) aortic valve implantation (TAVI) on platelet count. They observed that platelet count systematically decreased after TAVI, with an average decrease of 34±15%, and a decrease in platelet count reported to be associated with in-hospital major adverse cardiovascular events and strongly influenced patient outcome. In the setting of TAVI procedures platelet activation can be caused by endothelial damage caused by prosthesis implantation, fibrinogen binding on metallic armatures, and shear stress modifications due to prosthesis implantation^[13]^.

Van Straten et al.^[14]^ compared thrombocytopenia after AVR with mechanical and biological valves. They concluded that patients undergoing AVR with the Carpentier-Edwards Perimount bioprosthesis or a Medtronic Freestyle stentless bioprosthesis had a lower minimum platelet count within the first five POD, compared to patients receiving ATS and St. Jude Medical mechanical prostheses.

In our study, the drop-in platelet count started to occur during the 3^rd^ POD with very slow recovery toward the 7^th^ to 10^th^ POD. The lowest drop in platelet count was down to 22% of the preoperative baseline level and occurred in patients implanted with the Perceval valve. As shown in other studies, there were no other clinical consequences associated with thrombocytopenia that was transient but resulted in more transfusion requirements.

The bovine pericardium leaflets of both Perceval S valve and Mitroflow bioprosthesis are fixed in a process using gluteraldehyde. The Mitroflow prosthesis is stored in a solution of gluteraldehyde and needs rinsing before it is used. On the other side, the Perceval S prosthesis and Freedom Solo prosthesis are detoxified with homocysteic acid to eliminate any residual aldehyde and then stored in an aldehyde free solution; so, it does not require rinsing prior to use. Homocysteic acid can have a damaging effect on vessel endothelial cells and can precipitate platelet aggregation resulting in both thrombocytopenia and thrombotic complications^[15]^. These valves are carefully washed after detoxification with a homocysteic acid-free solution and then stored in jars filled with a homocysteic acid-free storage solution. Consequently, the residual amount of homocysteic acid, which could be transferred to the patient during the valve implantation, is negligible and the resulting concentration in blood is extremely low, so cannot be blamed for the postoperative thrombocytopenia. Moreover, the same solution is used for other bioprosthesis^[16]^, without causing higher incidence of thrombocytopenia.

Mechanical stress and hemodynamic turbulence have also been proposed as a cause of thrombocytopenia after Freedom Solo prosthesis^[10]^.

Small valve sizes have been described as causing some turbulence across the valve resulting in platelet activation or destruction, and consequently postoperative thrombocytopenia^[7,9,14]^. However, sutureless valves are well known for their superior hemodynamic performance and this hypothesis is quite unlikely.

Non-specific activation of platelets leading to diffuse consumption and thrombocytopenia after AVR with both mechanical and bioprosthetic aortic valves was suggested by Leguyader et al.^[17]^. Platelet activation could also result from valve manipulation or the presence of the metal stent.

The Perceval S valve has metal structure that serves as an anchoring system to the aortic valve, which could be the cause of turbulent flow, platelet rupture and mechanical structure. The metal stent may play a role in platelet activation and the possibility of paravalvular leaks may also trigger platelet activation and consumption. Moreover, the metal stents of these valves could interfere with the quality of images and could conceal or underestimate the degree of some paravalvular leaks^[5]^.

Long CPB duration has been suggested by other studies to be associated with postoperative thrombocytopenia^[14]^. In our study the CPB durations were shorter in the sutureless group than the sutured one so it cannot be responsible for post-operative thrombocytopenia.

Severe thrombocytopenia is a common complication in patients who undergo cardiac surgery with ECC. It occurs as result of structural changes and activation of platelets, hemodilution, platelet aggregation and bleeding^[18]^.

### Limitations

The main limitation of our study is due to its observational, non-randomized and retrospective characteristics. Heparin induced thrombocytopenia was not investigated as a possible cause of thrombocytopenia, as it appears after the 5^th^ postoperative day^[18]^.

## CONCLUSION

It seems to be an association between sutureless Perceval valve and severe thrombocytopenia, although it does not affect patient's mortality and morbidity. These patients had also higher rate of blood and platelets transfusion. A prospective randomised trial is needed to confirm our findings.

**Table:** 

Authors' roles & responsibilities	
SSM	Drafting the work or revising it critically for important intellectual content; final approval of the version to be published
SL	Drafting the work or revising it critically for important intellectual content; final approval of the version to be published
ARS	Drafting the work or revising it critically for important intellectual content; final approval of the version to be published
SS	Drafting the work or revising it critically for important intellectual content; final approval of the version to be published
SC	Drafting the work or revising it critically for important intellectual content; final approval of the version to be published
TP	Drafting the work or revising it critically for important intellectual content; final approval of the version to be published
